# The well now course: a service evaluation of a health gain approach to weight management

**DOI:** 10.1186/s12913-021-06836-z

**Published:** 2021-08-30

**Authors:** Fiona Clarke, Daryll Archibald, Valerie MacDonald, Sara Huc, Christina Ellwood

**Affiliations:** 1 Community Dietetics, RNI, Ness Walk, Inverness, IV3 5SF UK; 2 School of Health Sciences, University of Dundee, 11 Airlie Place, Dundee, DD14HJ UK; 3 Health Improvement Team, Public Health, NHS Highland, Larch House Stoneyfield Business Park, Inverness, IV2 7PA UK; 4 David Anderson Building, Aberdeen Royal Infirmary, Foresterhill, Aberdeen, AB25 2ZP UK

**Keywords:** Public health, Obesity, Healthy weight, Non-diet approach, Service evaluation, Health at every size (HAES)

## Abstract

**Background:**

The Well Now health and weight course teaches body respect and health gain for all. The course validates peoples’ lived experiences and knowledge through group activities and discussion with the aim of helping people to better understand their food and body stories. Well Now explores different ways of knowing, including the use and limits of body signals, like energy levels, hunger, taste and emotions and helps people keep food and behaviours in perspective by drawing attention to other factors that impact on health and wellbeing. This study undertook a service evaluation of the Well Now course to understand its acceptability for participants and its impact on diet quality, food preoccupation, physical activity and mental wellbeing.

**Methods:**

This service evaluation combined quantitative pre- and post-course measures with telephone interviews with previous attendees. Paired t-tests were used to determine if there were statistically significant differences in the intended outcomes. Semi-structured qualitative telephone interviews were undertaken with previous attendees 6–12 months after attendance to understand how participants experienced the Well Now course.

**Results:**

Significant improvements were demonstrated in diet quality, food preoccupation, physical activity and mental wellbeing outcomes. Medium effect sizes are demonstrated for mental wellbeing and diet quality, with smaller effect sizes shown for physical activity and food preoccupation. The weight and Body Mass Index (BMI) of attendees remained stable in this timeframe. The qualitative data corroborates and extends elements of the quantitative outcomes and highlights areas of the course that may benefit from further development and improvement. The findings further indicate that the Well Now approach is largely acceptable for attendees.

**Conclusions:**

Well Now’s non-judgemental holistic approach facilitates change for those who complete the course, and for those who do not. This health gain approach upholds non-maleficence and beneficence, and this is demonstrated with this service evaluation for both completers and partial completers.

**Supplementary Information:**

The online version contains supplementary material available at 10.1186/s12913-021-06836-z.

## Background

Public health messages linking food, activity, health and weight can be delivered within 3 key frameworks: diet, non-diet and health justice. Traditionally, services across the UK have relied on a diet paradigm and this is reflected in one of the six Public Health priorities for Scotland [1] that of having a ‘healthy weight and enjoying being physically active’. This paradigm also shapes the Scottish Government’s 2018 document, A Healthier Future: Scotland’s Diet and Healthy Weight Delivery Plan [2], which details a vision for improving health across Scotland including via outcome three, ‘*access to effective weight management services’*.

The diet approach can however oversimplify the relationship between lifestyle, health and weight, and ignore the impact that non-lifestyle factors have on health. It also, often equates following diet and activity guidelines with achieving a ‘healthy weight’. It presupposes that a strong evidence base exists to demonstrate the benefits of attempted weight loss are improved health, and that there are no adverse effects to this process.

The above stated Scottish Government strategy [2] acknowledges the impacts of both health inequalities and size stigma on the physical and psychological health of people living with high BMI. This could be seen as mitigating some of the problems with a diet (weight centric) approach. However, the focus on weight loss services may be counterproductive as there is a paucity of evidence to demonstrate the long-term effectiveness of intentional weight loss [3] whilst the focus on thinness as a route to health can potentially reinforce weight stigma [4].

National guidelines to implement and deliver weight management interventions involve multicomponent behavioural interventions to promote weight loss and thereby improve health [5, 6]. However, weight loss focused interventions demonstrate only a modest impact on weight overall, whereby weight loss maintenance is limited [7] and weight regain is common [5]. Community programmes vary in intensity, but interventions do not achieve the recommended 5% weight loss outcome for the majority of people who start the programme [8–10] and weight loss outcomes diminish as time post programme increases [8, 11, 12] . These findings are also supported by a population based cohort study which examined the primary care electronic records of over 176,000 adults with a high BMI, and found that the probability of attaining or maintaining weight loss is low, concluding that treatment grounded in community-based weight management framework may be ineffective [13].

Despite the ubiquity of a weight centric approach there is little evidence to support the premise that weight loss will lead to improvements in health. For example, systematic reviews of randomised control trials of weight loss interventions that report on health outcomes concluded that there were minimal effects on health outcomes (improved cholesterol, triglycerides, blood pressure, fasting glucose), and that the few positive effects (reductions in the use of hypertensive and diabetes medication) were not correlated with weight change [4, 14]. In addition, a predominant focus on weight loss may also foster weight stigma, which has adverse effects on people’s health and wellbeing, including: feelings of worthlessness and loneliness; depression, anxiety and other psychological disorders; stress-induced pathophysiology; avoidance of medical care [15]. Studies that claim to demonstrate the health benefits of weight loss services appear to share one or all of the following shortcomings: small numbers; short duration; do not report adverse effects; take no account of life circumstances [16].

One of the alternatives to a diet approach is a non-diet approach [17–19]. Characteristics of non-diet approaches are that they view health and wellbeing as multifaceted, direct efforts to improve health, improve access to services, and decrease size stigma. A review of non-diet approaches concluded that participants made sustained changes in practices that improve both physical and psychological health over time [17], and argued for more of these approaches to be undertaken and evaluated. A further review of non-diet approach trials [18] found that when compared with weight loss approaches, non-diet approaches achieve better physiological (e.g. blood pressure, blood lipids), health behavioural (e.g. eating and activity habits, dietary quality) and psychological (e.g. self-esteem and body image) outcomes for participants. This review further developed guidelines for non-diet approaches that recommend approaches should promote self-esteem, convey that lifestyle behaviours have limited impacts on health outcomes, avoid using language that evoke weight-based stigma such as overweight and obesity, and should have a compassion centred approach [18]

**Fig. 1:**
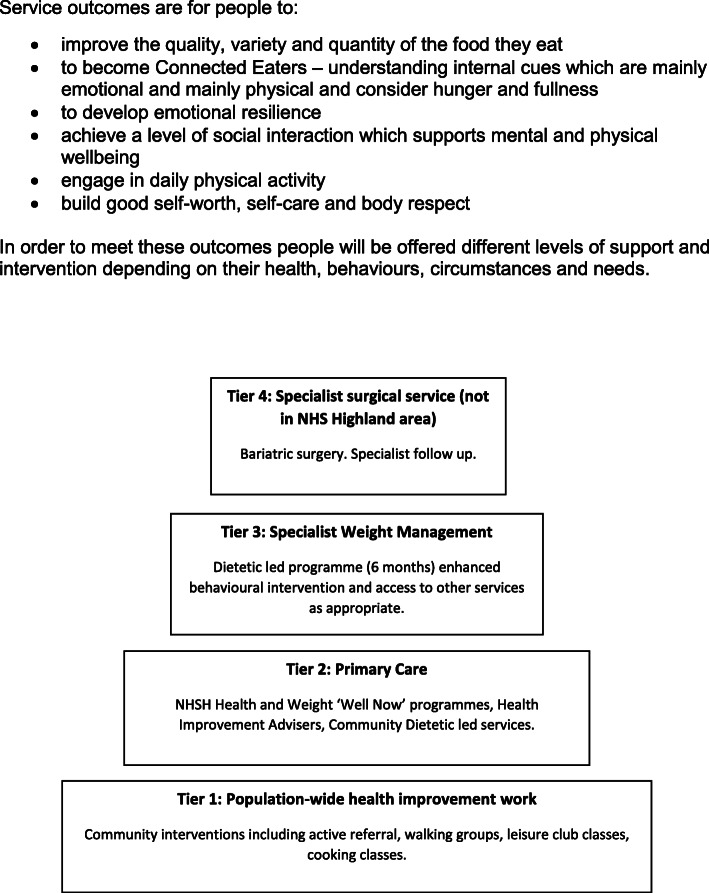
NHS Highland (NHSH) Adult Health and Weight Pathway - outcomes

These guidelines have influenced a health justice approach undertaken by NHS Highland (NHSH) within their health and weight services since 2013. The pathway aligns with the Scottish Government stepped approach (Fig. 1) to delivery. Service specific outcomes include improvements in lifestyle, enhanced mental and physical health and reduction in weight stigma. As with non-diet, a key element of the approach is that the focus of intervention is not centred on weight loss as a goal in itself but rather the improvement of health and/or wellbeing. One difference between the non-diet and health gain approaches is that the latter recognises the impact of life circumstances on health and weight, as well as health behaviours, and this is incorporated in the delivery of services.

At a Tier 2 community level these interventions have been delivered through the Well Now course. Well Now was designed by Registered Dietitian Lucy Aphramor and teaches body respect and health gain for all. It acknowledges a broad definition of health, and the impact that being treated with respect has on health and wellbeing. The course validates peoples’ lived experiences and knowledge through group activities and discussion with the aim of helping people to better understand their food and body stories, leading to an enhanced sense of coherence. The exercises explore different ways of knowing, including the use and limits of body signals, like energy levels, hunger, taste and emotions and helps people keep food and behaviours in perspective by drawing attention to other factors that impact on health and wellbeing.

This course is delivered in a group-based format by trained and licensed facilitators, usually over 6 weekly 2-h sessions. Facilitators receive training on the approach which is delivered in a group setting over 4 days. There is a post course and an annual assessment. New facilitators are offered the option to co-deliver Well Now groups with an experienced practitioner until they feel confident in both the approach and facilitation. Adults can access the course though self-referral or are referred into the dietetic services and offered interventions according to need.

This paper presents the findings from a service evaluation for this health gain approach when delivered in communities. The objectives of the study were to quantitatively measure to what extent participants meet the outcomes defined by the course at completion and, to qualitatively evaluate the course approximately 6–12 months following attendance.

## Methods

This was a service evaluation that combined quantitative pre- and post-course measures with qualitative telephone interviews with previous attendees. The evaluation was registered with the Clinical Audit Department of NHS Highland and was deemed exempt from review by the NHS ethics committee at NHS Highland. It is recognised that the boundaries between service evaluation and research can be a grey area, with Chen and Fawcett (2017) [20] arguing that the main ethical concern is wrongly labelling enquiry as research and non-research activities. This project can be described as a service evaluation which is ‘designed and conducted solely to define or judge current care’ [21] and is congruent with best-practice ethical principles such as consent, anonymity, data protection and privacy of patient [22], as is the Well Now philosophy.

An information sheet about the quantitative and qualitative elements of the study was given to all prospective participants. It was made clear to prospective participants that they could withdraw at any time without having to give an explanation. Procedures involved in the study were explained in the information sheet. Prospective participants were asked to provide verbal consent to take part in the study. All prospective respondents for the qualitative interviews were supplied with the study information sheet at least 24 h before the interview took place.

### Quantitative data

#### Participants

All Well Now course attendees that had consented to their data being used for the purposes of evaluation between April 2015 and December 2018 were included in the analysis.

#### Measures

Attendees were asked to complete the following outcome measures at the first and last session of the course.

- A 6-point Well Now questionnaire: assesses self-reported intakes of target foods over the past 7 days measuring changes in diet quality and quantity (a non-validated measure developed for the course).
- The Scottish Physical Activity Screening Question (available from: http://www.paha.org.uk/Resource/scottish-physical-activity-screening-question-scot-pasq)^:^ is a single validated question measuring changes in physical activity [23]
- Food Preoccupation Questionnaire (FPQ) [24]: a validated 3-part questionnaire which assesses frequency of thoughts about food (including positive and negative thoughts).
- Warwick Edinburgh Mental Wellbeing Scale (WEMWBS) questionnaire [25]: a validated questionnaire measuring changes in mental wellbeing:

The following information was also collected wherever possible for monitoring purposes:

- Height and weight at session 1, and then weight again at session 6, so that weight and BMI change can be calculated. Weight was collected using SECA 899 portable scales and height on SECA 213 portable stadiometer. Both measures were taken by the facilitators who had received training on the procedures, and the participants were in their indoor clothing, no footwear.
- Equality and Diversity data via a questionnaire in order to monitor the reach of the service.
- The Scottish Index of Multiple Deprivation (SIMD) [26] identified using attendee’s postcode. The SIMD is a validated area-based measure of socioeconomic circumstances (income and benefits; employment; health; education; access to services and transport; crime rates; housing). The SIMD ranks data zones across Scotland each containing approximately 750 people. These are categorised into quintiles where 1 is the most deprived and 5 is the least deprived.

#### Analysis

Analysis was performed using Microsoft Excel (2010) [27] and baseline data of the attendees was examined using descriptive statistics. Paired t-tests were used to determine if there were statistically significant differences in the intended outcomes and the effect size was calculated to provide an indication of magnitude of the change. Outcome data was compared between those who attended the required 75% or more of the course that categorised them as ‘complete’ and those who attended less than 75% that categorised them as ‘partial’ using a single factor Analysis of Variance (ANOVA).

### Qualitative data

To understand how participants experienced the Well Now course semi-structured qualitative telephone interviews were undertaken with previous attendees 6–12 months after attendance. The aim of these interviews was to collect views on the structure, content and approach of the group course, explore any health-related changes or other outcomes participants attributed to attending, and impact beyond the end of the course.

#### Participants

**Fig. 2:**
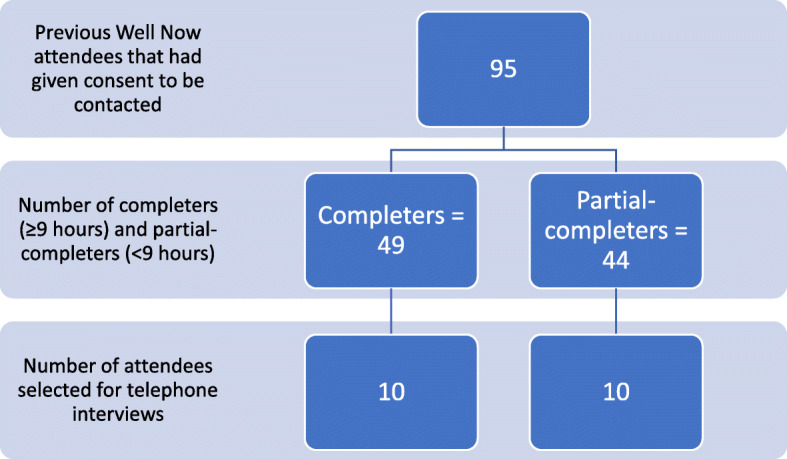
Sampling procedure for telephone interviews

Informed consent to be contacted for the purpose of service evaluation and improvement was sought when participants attended the Well Now sessions. Figure 2 shows the sampling procedure that resulted in 20 previous attendees being contacted to request feedback. A systematic method of sampling was used selecting every 4th attendee from the sampling frame.

**Fig. 3:**
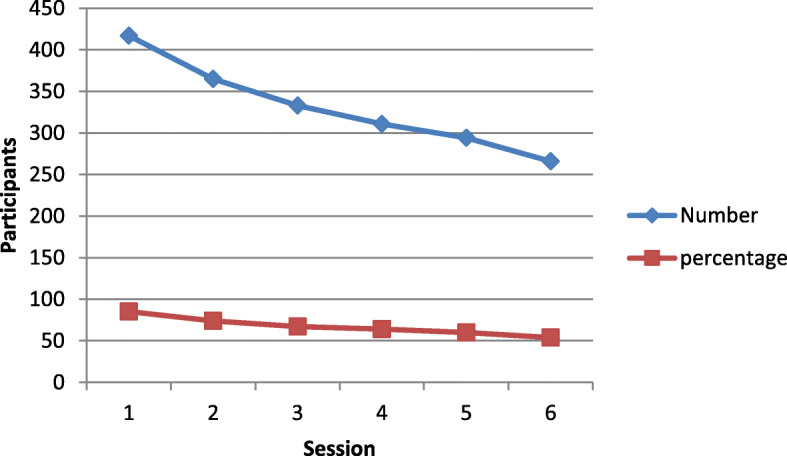
Attendance trend for participants attending the 6-session course (number and percentage)

#### Procedure

Potential participants were telephoned to ask if they would be interested in taking part in the interview phase. The nature and purpose of the interview was explained and assurances of confidentiality, anonymity, data protection and option to withdraw were provided. Nineteen participants provided verbal consent to continue. The interviews were conducted by FC and VM (Health Improvement Specialists, both trained in Motivational Interviewing) and were digitally recorded and transcribed verbatim. Transcripts were examined for inconsistencies against the recordings and anonymised. The interview schedule can be found in Appendix 1. The characteristics of the qualitative sample can be viewed in Appendix 2.

#### Analysis

The transcripts were analysed using thematic analysis [28]. In the first instance, transcripts were read and re-read individually. The data were then matched to categories outlined in the semi-structured interview guide. A coding scheme that formed the building blocks of the thematic analysis was then developed inductively. Themes were then generated by FC and DA after reading the coded data. These were discussed and refined until consensus was reached and reviewed by CE.

## Results

### Quantitative analyses

Five hundred and thirty-seven people joined the Well Now course between April 2015 and December 2018, two people did not consent to their data being stored. People’s demographic, equality and diversity profiles are presented in Table 1. Data is presented for all those who agreed to complete or partially complete the questionnaires. Three quarters of attendees were female, with the largest proportion of attendees falling into the 40–64 years age group. There was an even spread of attendees from all socioeconomic areas (as categorised by the SIMD), the largest proportion (21%) were from the areas categorised at experiencing the highest levels of multiple deprivation. Over half of the attendees experienced a long-term health condition, with one quarter reporting a mental health condition and almost 40% reporting problems with mobility.

**Table 1:** Baseline characteristics of attendees who commenced a Well Now course between April 2015 – December 2018

	Participants (*n* = 535)	% of Participants
Sex		
Male	96	18
Female	395	74
unknown	44	8
Age Band (years)		
Under 16	0	0
16–24	13	2
25–44	131	25
45–64	219	40
65 & over	94	18
Unknown	78	15
SIMD		
1 (most deprived)	114	21
2	91	17
3	40	7
4	90	17
5 (least deprived)	90	17
Not known	110	21
Equality and Diversity	**(*n*** **= 447)**	
Presence of physical and/or mental health condition	262	59
Health condition reduces ability to carry out day-to-day activities:		
a lot	110	25
a little	118	27
Ethnicity:		
White	427	96
Asian	1	< 1
African	1	< 1
Caribbean or Black	1	< 1
Other	3	1
Unknown	14	3
Religion		
None	135	30
Church of Scotland	135	30
Roman Catholic	27	6
Other Christian	63	14
Muslim	1	< 1
Buddhist	4	1
Pagan	2	< 1
Other	7	2
Prefer not to answer	24	5
Unknown	49	11
Sexuality		
Heterosexual/straight	387	87
Gay/Lesbian	11	3
Bisexual	4	1
Other	1	< 1
Prefer not to answer	9	2
Unknown	35	8

#### Attendance and attrition

Sixty-five Well Now courses were delivered over the period. The course content of 12 h was either delivered over six or eight sessions depending on facilitator and venue availability, with 492 and 45 people enrolling on each, respectively. In all cases the first session had the highest attendance (86% of confirmed referrals) and this gradually reduced throughout the course (59% in the last session for both versions), mean attendance was 7.5 out of 12 h (Fig. 3). Further information on attrition by sex, age and SIMD status is available in appendices 3 and 4.

#### Pre and post course measures

The primary outcomes for the course are changes in diet quality and food preoccupation, physical activity, and mental wellbeing. Participants had to provide answers to all questionnaire questions for their responses to be considered valid [24, 25]. Between 47 and 56% of attendees fully completed measures at both time points and were included in the analysis. There were statistically significant improvements in all intended outcomes (Table 2). Mean WEMWBS scores increased from 45 (range 12 to 68) to 51 (range 14 to 70). The Scottish average is 50 and 51 in Highland [29]. Mean Well Now scores increased from 20 (range 3 to 33) to 23 (range 6 to 36). The ‘ideal’ score is 29 in that it constitutes an intake that meets current population recommendations [30]. Mean physical activity scores increased from 2.7 (range 0 to 7) to 3.7 (range 0 to 7) days per week. Guidelines encourage physical activity for 30 min between 5 and 7 days per week [23]. Mean FPQ frequency decreased from 10 (range 3 to 15) to 9 (range 2 to 15). Mean FPQ positive thoughts increased from 27 (range 9 to 45) to 28 (range 9 to 45). Mean FPQ negative thoughts decreased from 24 (range 9 to 45) to 22 (range 9 to 45).

**Table 2:** Changes in pre and post course questionnaire scores for all participants, using paired t-test

Measure	Number (%) of Participants	Difference in Score (Pre vs. Post)			*p*-value^1^	Effect size^2^
		Mean	Lower CI (95%)	Upper CI (95%)		
WEMWBS	301 (56)	5.85	4.86	6.83	< 0.01	0.68
Well Now - diet quality	290 (54)	3.40	2.81	4.0	< 0.01	0.66
Physical activity	282 (53)	1.01	0.76	1.26	< 0.01	0.47
Pre-occupation with food - frequency of thoughts	254 (47)	−0.70	−1.08	−0.31	< 0.01	− 0.22
Pre-occupation with food - positive thoughts	254 (47)	1.16	0.27	2.05	< 0.01	0.16
Pre-occupation with food - negative thoughts	254 (47)	−2.48	−3.34	−1.62	< 0.01	−0.36

^*1*^ *< 0.01 = significantly different at 1% level,* ^*2*^ *= Cohen’s d ranges (0.2- small, 0.5 – medium, 0.8 – large)*

Weight loss is not an intended outcome of the course and weight stability would be expected. There was a small statistically significant change (*p* < 0.05) in weight throughout the 6–8-week course in the 129 people who consented to pre and post course weights (− 0.4 kgs). The effect size was very small at 0.18 which indicates that the course did not necessarily have an effect on weight, and that it could have been due factors out with the course.

To examine whether full attendance was associated with significantly more change in targeted outcomes a single factor ANOVA was undertaken comparing those attendees that completed a ‘full’ course to those that complete a ‘partial’ course. The results are presented in Table 3. Those that attended the full course reported a significantly higher increase in mental wellbeing than those who attended a partial course. For all other measures there was no significant difference in outcome.

**Table 3:** Comparison of outcomes for those who had completed the full course (≥9 h). and those who had partially completed (< 9 h) the course

Measure	Mean change		*p*-value^1^
	Complete	Partial	
WEMWBS	**6.47**	**4.16**	**< 0.05**
Well Now - diet quality	**3.67**	**2.70**	**NS**
Physical activity	**1.02**	**0.96**	**NS**
Pre-occupation with food - frequency of thoughts	**−0.84**	**− 0.27**	**NS**
Pre-occupation with food - positive thoughts	**1.92**	**0.88**	**NS**
Pre-occupation with food - negative thoughts	**−2.66**	**−1.93**	**NS**

^1^ < 0.05 significant at the 5% level, *NS* Not significant

### Qualitative data

The qualitative analysis generated seven themes. These were: *Effective and Affirmative Facilitation, Impact of the Group Context, Perceived Benefits, Dietary and Physical Activity Change, Social Connection, Self-Compassion and Acceptance, What Well now means.* These are presented below.

#### Effective and affirmative facilitation

Establishing rapport and building a safe environment is integral to the Well Now ethos. Although the courses were delivered by different facilitators depending on time and location, the facilitation was consistently commented on positively. Participants described the facilitators as friendly, encouraging and knowledgeable. The inclusive nature of the approach taken by facilitators was key for a number of attendees:

*“It was very friendly and didn’t make you feel guilty for being big. There wasn’t any … what’s the right word? Stigmatise you or make you feel guilty.*” (Partial attendee)

It was also noted that the facilitators worked to bring the group together and engaged the group well:

*“The facilitators knew the people that were coming in were all in the same boat, they didn’t know each other, and I think most of the first session was getting everybody at ease and going over what they were going to do. So, it was all done really well.”* (Complete attendee)

#### Impact of the group context

The group setting was helpful to some respondents; there was camaraderie, social support and a sense of relatedness:

*“The main thing was that everybody that was on had a similar reason for being there. We were part of a team and a very friendly place to go.”* (Complete attendee)

Others found it more of a challenge and felt uncomfortable in a group, or felt the group lacked cohesion:

*“Some people didn’t speak out at all, you know. Like we all sat in a circle or we all sat in chairs at different tables.”* (Complete attendee)

Comments about the logistics of the groups were overwhelmingly positive; groups varied in location and time, but the choices were appropriate and met the needs of the attendees.

*“We were part of a team and a very friendly place to go; I liked the whole thing generally; it was nice and varied each week.”* (Partial attendee)

#### Perceived benefits

There were a range of beneficial aspects to the course that were noted by respondents, notably the nutritional information and the supporting materials. People also mentioned the helpfulness of legitimising all food, addressing health and wellbeing more holistically, and generally being offered new information to consider.

*“So, I think for most people that is a really good way to tackle it from a health point of view and not to fixate. Better I know myself to be overweight and healthier and fitter than skinny people.”* (Complete attendee)

*“They just didn’t cover food. You know they just didn’t cover the things you should eat and the things you shouldn’t eat. There was a lot of different ways of thinking.”* (Complete attendee)

#### Dietary and physical activity change

The responses support the quantitative data that indicate attendees increase their diet quality during the group, a change that appears to be maintained in the following months. Thirteen participants of the twenty interviewed, reported that there has been a positive change in their diet; the majority of the comments talked about introducing new food and food they felt would benefit their health, reflecting the Well Now approach of moving away from restricting the diet to nurturing the body:

“*I’m sort of aware of now different pulses, wheatmeal, and wholegrain grains. Look more at the bread I buy and pasta and rice probably go more for the wholegrain and eat more fruit.”* (Partial attendee)

A core part of Well Now is encouraging attendees to attune to and respond to their body’s needs, particularly around hunger and fullness and recognising other reasons why they might eat other than hunger. A small number of respondents commented on making changes to how or why they ate, indicating that this element had been understood and retained by some:

*“Yes definitely, definitely that has changed. I stop eating when I’m full whereas before I ate from the clock. It was 1 o’clock it was lunchtime, 5 o’clock it was dinner time. Now it’s started to recognise when I’m hungry not when the time dictates.”* (Partial attendee)

Eight of the participants commented on changes to their levels of physical activity following the group:

*“Trying to walk a bit more.”* (Partial attendee)

Ten participants reported no change to their physical activity. This was often attributed to difficulties in their physical health and perceived barriers, or that they were already physically active:

*“Not as yet. My joints are very bad”* (Partial attendee)

*“I was doing that anyway. It’s the same. I’d already lost my weight and I try and do a wee bit of walking every day so … ”* (Complete attendee)

#### Social connection

The course encourages attendees to consider their social network and how this might be a factor in their overall wellbeing. Four respondents specifically commented on increasing their social activity:

*“We have actually taken up dancing.”* (Complete attendee).

“*now going out 3 times a week,” (Complete attendee).*

*“first time since 1999 my sister and family came up to XXXX and they had a spare (caravan) berth. Now normally I wouldn’t have gone for a week. I did go.” (Complete attendee)*

#### Self-compassion and acceptance

A key message in Well Now is the potential impact that being kinder to yourself and accepting yourself the way you are can impact positively on your wellbeing and encourage a more nurturing approach to self-care. Five respondents talked about a change in how they felt about themselves, or recognition that perhaps they had not been as kind to themselves as they deserved before:

*“I think I have accepted myself more and like myself a little bit more. Still not overly happy but it did have an impact*.” (Complete attendee)

#### What Well Now means

The reflections by respondents on the key messages of Well Now picked out a number of core elements of the course, which is encouraging. They identified a more holistic approach to wellbeing, the link between food and mood, respecting and responding to your body and what you need, acknowledging stigma, and self-acceptance:

*“Tuning in to your body and recognising if you were hungry and if you weren’t hungry and what you were feeding yourself. Just feeding another emotion.”* (Complete attendee)

“*Not to be hung up on your weight. Be more holistic, look at everything not just thinking of a diet, the whole, the everything rather than just your weight which was good because often things are just get the weight off and then concentrate on being healthy. It was more holistic.”* (Complete attendee)

However, the philosophy of Well Now did not always resonate with those that attended, and respondents expressed confusion or disagreement with some or all of the elements:

“*Everything about it was completely the opposite of my way of thinking.”* (Partial attendee)

*“To be honest I couldn’t understand it properly. What the gist of the thing was. What I needed at that time was somebody to say this is how you do it. You know. I just couldn’t quite get a feel of where it was going at that time.”* (Partial attendee)

This could be viewed as a potential harm of the Well Now approach, particularly if these feelings result in a participant attending less of the course. However, most people who commence the course display a degree of confusion regarding the approach, which is most likely due to expectations associated with the cultural norms of the traditional weight loss approach, and possibly, internalised weight stigma. People joined this NHS course expecting a weight normative approach, and some were concurrently actively engaged in weight normative interventions, thus it is likely difficult to then grasp a weight inclusive approach. We feel that rather than this being a fault of the programme, this is more indicative of social norms around weight normative approaches.

There were also comments made by the respondents about the key things they have taken from the course that raise the possibility of unintended outcomes.

*“The only thing that has changed for me that I restrain more of what I am eating*.” (Complete attendee)

*“I do kind of try to do more portion control.”* (Partial attendee).

Well Now does not aim to ‘fix’ peoples eating; it seeks to help people make sense of their experiences and reduce the impact of emotional distress. We do not know what participants mean by ‘restraint’ or ‘portion control’ - it might result in a reduction of binge eating/eating disorder symptomology – or it might not.

As well as gathering valuable feedback from attendees to the Well Now course as a whole we were interested in exploring whether perspectives differed between those that had completed all or most of the course (≥75% attendance) and those that had completed only a partial course (< 75%). Content analysis of the frequency of utterances by both groups within the themes identified found no notable differences (data not presented).

## Discussion

This paper aimed to evaluate an innovative approach to health and weight in NHS Highland using both quantitative and qualitative methodologies. The results indicated that the Well Now course, delivered by trained facilitators across the Highland Council area, supported attendees to make improvements to their diet and food preoccupation, physical activity, mental wellbeing.

### Course outcomes

Significant improvements were demonstrated in all targeted outcomes at course completion. Medium effect sizes were demonstrated for mental wellbeing and diet quality, with smaller effect sizes shown for physical activity and food preoccupation. Those that attended the full course reported a significantly higher increase in mental wellbeing than those who attended a partial course. This may be related to increased social support from the group, and/or may reflect circumstances and challenges that hinder some peoples’ attendance. The weight and BMI of attendees remained stable over the 6–8-week course. Although a mean − 0.4 kg weight change is statistically significant it is clinically insignificant. Monitoring weight is not a usual component of the course but was included as it is one of the Scottish Governments reporting outcomes. However, some people opted out of the end of course weigh-in as they appeared to eschew the value of using weight/weight change as a proxy for health, which is exemplified in the qualitative findings above regarding not “fixating” or being “hung-up” on one’s weight.

The evaluation purposefully included looking for adverse effects by using the food preoccupation questionnaire, WEMWBS and the qualitative interviews particularly with those who did not complete the course. It is important not to make exaggerated claims on the basis of any course of short duration, or to extrapolate results to imply longer term effects. However, this data is useful for a tendency for weight stability, improvements in a range of physical and mental health parameters, and importantly, the likely absence of adverse effect.

To our knowledge, the use of health justice approaches in weight and health services is new and there is no existing body of evidence to draw on, beyond earlier evaluations of Well Now in England. Our findings are consistent with a qualitative study where participants described how engaging with the Well Now philosophy in a supportive group had beneficially impacted their health and sense of self-worth [31]. The reorientation made available through Well Now enhanced psychosocial variables and behaviours known to impact on health, such as mood, self-esteem, eating/exercise habits and interpersonal relationships. Participants recounted instances where recommendations to follow a weight-corrective approach, and attendant size bias seen in health practitioner’s attitudes, had had a detrimental impact on their wellbeing and sense of self-worth [32].

### Non diet approaches

Direct comparison between Well Now and non-diet approaches is not possible, but there are elements which are similar: both advocate size acceptance and recognise and challenge the reliance of cognitive restraint in eating for wellbeing. Health gain approaches evolved from non-diet (including HAES®) approaches but differ in their pedagogy, language and social justice [32]. A study examining the effects of a HAES® intervention in a community-based healthcare context found significant increases in their diet quality scores as measured by the Healthy Eating Index and intuitive eating [33]. The control group of this study followed the traditional weight loss approach even though the adverse outcomes of dieting were anticipated. A 10-week intervention based on intuitive eating principles was undertaken with a military population [3] and found that intuitive eating scores increased without a clinically significant weight loss (> 5% body weight). It should be noted that Well Now promotes ‘connected eating’ as oppose to intuitive eating as this recognises the uses and limits of body signals [34].

The qualitative data corroborates and extends elements of the quantitative outcomes and highlights areas of the course that may benefit from further development and improvement. It indicates that the Well Now approach is largely acceptable for many attendees. The holistic nature of the course looking beyond diet and exercise to include the many other factors involved in wellbeing was noted as a particularly valuable aspect.

### Scope and reach of the course

Monitoring information collected in the Equality and Diversity questionnaire reflects a positive reach of the service; the qualitative data suggested that the impact of comorbidities influenced people’s ability to either continue attendance to a group or to implement intended changes following course completion. Further analysis of the attendance and attrition rates may provide insight into whether the course can facilitate continued attendance in those people with comorbidities. Sexual orientation data indicates that the proportion of attendees reflects the United Kingdom (UK) statistics for the different groups. There were lower numbers of men attending and this reflects national data on the use of weight management services by men. There is a need to develop the services to further engage men and recognise and engage people of all genders (Robertson et al. 2014) [35].

The socio-economic status of the attendees (as measured by the SIMD) represents attendance from all groups with a slightly higher proportion attending from the two groups categorised as experiencing the highest level of multiple deprivation. Targeting population groups with the most need is crucial in reducing health inequalities and further work needs to be undertaken to increase the proportion of these groups benefitting from the service.

### Limitations

There are a number of limitations within this evaluation that should be considered. Firstly, it is important to note that the non-experimental design of this study does not permit causality to be inferred. In addition, the completeness of the quantitative data was relatively low. Between 47 and 56% of referred attendees completed both pre- and post-course measures depending on questionnaire. This may have been due to attrition from the course i.e. not attending the sixth session in which post-course measures where completed, or declining to complete measures if in attendance. Although there were no differences in baseline characteristics between ‘completers’ and ‘partial-completers’ (data not presented), there may be important variables involved in attending a Well Now course and the outcomes of those who do not attend or continue to attend that we have not been able to capture. This inevitably introduces bias into the service evaluation in that we have reported on data for those who completed the evaluation measures. It also lacks information on clinical risk factors such as blood pressure, glycaemic control or changes in medications that would be beneficial in evaluating the impact on attendee’s overall health and wellbeing. However, collection of these measures is not appropriate in a real-life community delivered intervention particularly at a self-management focused level of the service.

A further limitation of the quantitative data is that the insights generated are relatively limited in their capacity to identify the ‘active ingredients’ of this particular course, why changes were made and what factors were involved. The qualitative data added to this picture, drawing out elements of the course that were of significance to the attendees, however those who took part in the telephone interviews were a small proportion of those that attended across the time period, and may not represent the views of all attendees. Another gap in this evaluation is that we did not enquire about how the course may have improved understanding in the wider determinants of health, and the science on weight and health. This omission risks representing the Well Now course as behaviour change intervention, when in fact it takes a community development approach to health. These could be areas of future investigation as the course continues to be implemented. A further limitation of the analysis can be observed in the heterogeneity of partial attendees. Some partial attendees may have missed sessions for logistical reasons whereby others may have consciously decided to stop attending. Future analyses of Well Now data could potentially treat these groups of partial attendees as separate sub-samples.

The main strengths of this study are that it is intended as an evaluation of public health services being delivered in communities; that it includes the views of partial as well as full completers and that it looked for adverse outcomes. The data is that of a population that access the service and so may be generalizable to populations in other geographical areas that utilise weight management services. The evaluation does not make assumptions of research study robustness; however, a particular weakness to consider when interpreting the results is the fidelity of the course delivery. There are a growing number of facilitators and delivery of the course is likely to vary, particularly as it is not intended to be a manualised course, but a values-based responsive intervention, with lesson plans to draw on. Facilitators undertake an annual assessment as part of the governance process. Clearly variations in facilitator knowledge and skills will influence participants’ experiences.

How does this leave Well Now in relation to priority 6 of Public Health Scotland [1] - that of having a ‘healthy weight and enjoying being physically active’? “Enjoying” physical activity concerns attitudes and behaviours, and having a particular weight is a characteristic, not a behaviour. We have focussed on behaviours and attitudes, and shown improvements in physical and mental health, with no adverse effects – which is the overall goal of the Healthier Scotland priorities and policies. Of note, participants also commented on the novelty and benefit of being offered a non-stigmatising service.

## Conclusion

This service evaluation adds to the evidence base reporting on the outcomes of Well Now, a health justice approach to health and weight, as delivered in communities [31, 32]. It reports on the views of both those who complete the intervention, and those who do not. Weight-centric approaches remain the prevalent paradigm despite the evidence that they do not provide health gains for the majority; potentially underestimate negative outcomes for people; ignore the social determinants of health (including trauma) and can potentially lead to size stigma. A health justice approach validates people’s lived experiences, including any impact of dieting and size discrimination. It was seen as non-judgemental and holistic which facilitated change for those who completed the course, and for those who did not. This health justice approach upholds non-maleficence and beneficence, and this is demonstrated with this service evaluation for both completers and partial completers.

**(Health at Every Size® and HAES® are registered trademarks of the Association for Size Diversity and Health).**

## Supplementary Information

**Additional file 1.**

**Additional file 2.** Qualitative sample.

**Additional file 3: Appendix 3**. Attendance by sex of participants attending the 6 sessions.

**Additional file 4: Appendix 4**. Starting Characteristics of those attending more or less than 9 h (completers and partial completers).

## Data Availability

the NHS Highland tier 2 database contains data that has been fully reported here but is not publicly available because it contains patient identifiable data. The data that support the findings in this study are available from the corresponding author upon request.

## Abbreviations

NHS: National Health Service
NHSH: National Health Service Highlands
BMI: Body Mass Index
LWMP: Liverpool Weight Management Programme
WEMWBS: Warwick Edinburgh Mental Health and Wellbeing Score
SIMD: Scottish Index of Multiple Deprivation
ANOVA: Analysis of Variance
FPQ: Food Preoccupation Questionnaire
HAES: Health at Every Size
UK: United Kingdom
