# Supplementary material for: The well now course: a service evaluation of a health gain approach to weight management
Source: BMC Health Serv Res. 2021 Aug 30;21:892. doi: 10.1186/s12913-021-06836-z (PMC8404319; doi:10.1186/s12913-021-06836-z)
Supplement: Supplementary file 1 — Additional file 1. [file 12913_2021_6836_MOESM1_ESM.docx]

**Well Now: Tier 2 groups based weight management evaluation**

**Telephone interviews:**

- Hello – introduce self
- Is this a good time to speak to you?
- Check name and dob.
- I’m calling to ask you a few questions about the Well Now group that you attended
- Firstly may I explain a bit about this:
  - You attended a Well Now course and at that time you agreed to be phoned so that you could tell us about how it was for you. It is your choice to take part or not. If you do agree to take part and then change your mind you can withdraw at any time
  - We are doing this to help us improve the service for people in the future.
  - The same questions will be asked to all interviewees.
  - This conversation is confidential. The findings will only be used in the evaluation of the service. They can be shared with those who take part. We may publish a report on this work.
  - We will be recording this conversation to make sure we get a true record of your views. We will then code the interviews, presenting all views anonymously. The recordings will be erased after use.

What questions do you have about this?

- It will take 10 to 20 minutes to go through – are you happy to continue?
- Thank you – your opinions & views are valued.

I’ll start with the first question……

- Use reflections and summaries throughout.

1. What did you like about the Well Now course?

*Prompts:* content

venue and the accessibility

style of facilitation and the people that ran it

length of the course was and the number of sessions

2. What, if anything did you not like about the course?

3. What, if anything has changed for you for you as a result of the programme?

*Prompts:* the way in which you eat

dieting behaviours

physical activity

going out more

accepting your body.

4. Can you tell me if there has been any impact on your weight since the end of the course?

5. If I was to ask you to give me a mark out of 10 as to how helpful the programme was, when 0 was absolutely no help at all, and 10 was of ultimate help. What score would you give?

6. And what where the key messages?

7. Within the course we use a few tools to help get the message across. Which of these:

do you remember?

continue to use?

8. Were there any surprises in the course?

9. What, if any, new behaviours have you adopted?

10. How might we do better? How might the service be improved?

11. Anything else in terms of improvement?

12. Is there anything else you would like to share with me about the service?

Finish with:

- Would you like to receive a summary of our findings? (check details)
- Can we contact you again at a later date; say in 6 or 12 months time?
- Thank you so much for your time today and participating in the evaluation, it’s been useful talking to you.
- Bye.
