# Supplementary material for: The well now course: a service evaluation of a health gain approach to weight management
Source: BMC Health Serv Res. 2021 Aug 30;21:892. doi: 10.1186/s12913-021-06836-z (PMC8404319; doi:10.1186/s12913-021-06836-z)
Supplement: Supplementary file 2 — Additional file 2. Qualitative sample. [file 12913_2021_6836_MOESM2_ESM.docx]

Qualitative sample

Nineteen of the twenty people gave consent and completed the qualitative evaluation. Fifteen (74%) identified as female and 4 (21%) as male; 74:17 in main cohort. Eleven (58%) completed the course and 8 (42%) partially completed; 50:50 in main cohort.

|  | Qualitative Participants (n=19) | % of Qualitative Participants | Participants (n=537) | % of Participants |
| --- | --- | --- | --- | --- |
| Sex |  |  |  |  |
| Male | 4 | 21 | 96 | 17 |
| Female | 15 | 74 | 397 | 74 |
| unknown | 0 | 0 | 44 | 8 |
| Age Band (years) |  |  |  |  |
| Under 16 | 0 | 0 | 0 | 0 |
| 16 – 24 | 0 | 0 | 13 | 2 |
| 25 – 44 | 3 | 16 | 131 | 25 |
| 45 – 64 | 7 | 37 | 219 | 40 |
| 65 & over | 4 | 21 | 94 | 18 |
| Unknown | 0 | 0 | 80 | 15 |
