# Supplementary material for: The well now course: a service evaluation of a health gain approach to weight management
Source: BMC Health Serv Res. 2021 Aug 30;21:892. doi: 10.1186/s12913-021-06836-z (PMC8404319; doi:10.1186/s12913-021-06836-z)
Supplement: Supplementary file 3 — Additional file 3: Appendix 3. Attendance by sex of participants attending the 6 sessions. [file 12913_2021_6836_MOESM3_ESM.docx]

**Appendix 3: Attendance by sex of participants attending the 6 sessions (percentage of total n=537))**
