# Supplementary material for: The well now course: a service evaluation of a health gain approach to weight management
Source: BMC Health Serv Res. 2021 Aug 30;21:892. doi: 10.1186/s12913-021-06836-z (PMC8404319; doi:10.1186/s12913-021-06836-z)
Supplement: Supplementary file 4 — Additional file 4: Appendix 4. Starting Characteristics of those attending more or less than 9 h (completers and partial completers). [file 12913_2021_6836_MOESM4_ESM.docx]

**Appendix 4: Starting Characteristics of those attending more or less than 9 hours (completers and partial completers)**

|  | Completers n=271 (50%) | | | Partial completers n=266 (50%) | | |
| --- | --- | --- | --- | --- | --- | --- |
|  | Number (%) with measure | Mean | Range | Number (%) with measure | Mean | Range |
| Male | 46 (17) |  |  | 50 (19) |  |  |
| Female | 210 (77) |  |  | 187 (70) |  |  |
| Unknown | 15 (6) |  |  | 29 (11) |  |  |
| SIMD 1+2 | 104 (28) |  |  | 101 (38) |  |  |
| BMI (kg/m^2^) | 132 (49) | 38 | 21.3-62.2 | 82 (31) | 39 | 15.5-61.7 |
| WEMWBS | 238 (88) | 44.7 | 17-68 | 197 (74) | 45 | 15-68 |
| Well Now | 234 (86) | 20 | 3-33 | 190 (71) | 19.3 | 1-33 |
| Physical Activity | 242 (89) | 2.7 | 0-7 | 207 (78) | 2.7 | 0-7 |
| FPQ frequency | 221 (82) | 10.3 | 3-15 | 187 (70) | 10 | 3-15 |
| FPQ positive thoughts | 209 (77) | 26.9 | 9-45 | 181 (68) | 26.5 | 9-44 |
| FPQ negative thoughts | 213 (79) | 25.2 | 9-45 | 183 (69) | 24.3 | 9-45 |
